# An outbreak of hepatitis A associated with salted clams in Busan, Korea

**DOI:** 10.4178/epih.e2022003

**Published:** 2021-12-29

**Authors:** Hyunjin Son, Miyoung Lee, Youngduck Eun, Wonseo Park, Kyounghee Park, Sora Kwon, Seungjin Kim, Changhoon Kim

**Affiliations:** 1 Department of Preventive Medicine, Dong-A University College of Medicine, Busan, Korea; 2 Busan Center for Infectious Disease Control and Prevention, Pusan National University Hospital, Busan, Korea; 3 Korea Disease Control and Prevention Agency, Cheongju, Korea; 4 Department of Preventive Medicine, Pusan National University College of Medicine, Busan, Korea

**Keywords:** Hepatitis A, Salted clams, Food-borne outbreak, Korea

## Abstract

**OBJECTIVES:**

In July 2019, there were multiple reports on patients with hepatitis A among the visitors of a restaurant in Busan. The current study presents the results of an epidemiological investigation and outlines the supplementary measures that would help with hepatitis A control.

**METHODS:**

A cohort study was conducted for all 2,865 customers who visited restaurant A from June to July. Using a standardized questionnaire, participants reported the presence of hepatitis A symptoms and whether they had consumed any of 19 food items. As for participants who had visited public health centers, their specimens were collected.

**RESULTS:**

From the study cohort, 155 participants (5.4%) had confirmed hepatitis A. The epidemic curve was unimodal, and the median number of days from the restaurant visit to symptom onset was 31 days. A genotype analysis indicated that 89 of 90 tested patients had hepatitis A virus (HAV) genotype 1A. The results of a multivariate logistic regression analysis indicated that the ingestion of salted clams increased the risk of hepatitis A by 68.12 times (95% confidence interval [CI], 9.22 to 510.87). In an unopened package of salted clams found and secured through traceback investigation, HAV genotype 1A was detected.

**CONCLUSIONS:**

To prevent people from ingesting uncooked clams, there needs to be more efforts to publicize the dangers of uncooked clams; the food sampling test standards for salted clams should also be expanded. Furthermore, a laboratory surveillance system based on molecular genetics should be established to detect outbreaks earlier.

## INTRODUCTION

The hepatitis A virus (HAV) is a ribonucleic acid virus in the *Picornaviridae* family, with humans as its only natural reservoir [1]. The main hepatitis A symptoms include fever, fatigue, loss of appetite, abdominal pain, and jaundice. Symptom severity increases with age and about 70% of infants aged < 6 years are asymptomatic. The hepatitis A vaccine is high effective if administered within 2 weeks of HAV exposure and can be prophylactic if administered after exposure [2].

The HAV is usually transmitted by the fecal–oral route through contaminated water, food, or close contact with an infected person. In nations with low endemicity, there have been reports of hepatitis A outbreaks that have occurred through various situations and foods. Hepatitis A outbreaks occur in medical facilities provide care for critically ill patients, elderly patients, and children [3,4] and in locations with poor hygienic conditions [5]. With regard to food items, there have been outbreak reports associated with unheated of insufficiently heated clams [6], frozen fruits [7], semi-dried fruits [8], and vegetables [9].

In Korea, between 2011 and 2018, the year 2011 had the largest number of reported hepatitis A cases with 5,521, while 2013 had the lowest number of reported cases with 867 [10]. In 2019, there were 17,598 reports of hepatitis A. Specially on June 25 and July 26, 2019, the Korea Disease Control and Prevention Agency (KDCA) reported that salted clams were the source of infection for some of small-scale hepatitis A clusters [11,12]. However, this was an insufficient explanation for the cause of rapid increases compared to the same period in the previous year, being more than 6 times.

In the spring of 2019, the number of patients with hepatitis A was rapidly increasing in the regions of Daejeon, Chungcheong Province, and the Seoul Metropolitan area. In Busan, until early July, there were approximately 5 weekly cases of hepatitis A, which was similar to the rate during other years. Then, during the second week of July 2019 (July 6 to 12), hepatitis A cases increased to 9 cases, and to 18 cases during the third week of July (July 13 to 19). Inspecting the spatial aggregation of cases reported during the second and third weeks of July, eight patients had been reported in district B, one of the 16 districts of Busan city. Following that weekend, there were 15 cases of hepatitis A on the single day of July 22. In-depth interviews were conducted with these 15 patients. It was found that 12 patients of 15 patients had visited restaurant A located in district B during June 2019. Therefore restaurant A was suspected to be associated with the outbreak. To ascertain the source of infection and prevent further transmission of the disease, the city of Busan and the Busan Center for Infectious Disease Control and Prevention established an epidemiological investigation team on July 23 and engaged in a comprehensive epidemiological investigation.

## MATERIALS AND METHODS

### Case definition and case finding

The case definition was as follows: among all people who visited restaurant A from June 1 to July 28, 2019, people with one or more hepatitis A symptoms (fever, fatigue, jaundice, abdominal pain, and vomiting) that occurred within 15-50 days of the visit, who had positive test results from the serum immunoglobulin (Ig)M anti-HAV antibodies test, or reverse transcription polymerase chain reaction (RT-PCR) test.

The cohort included all customers who had paid at restaurant A by credit card between June 1 to July 28, 2019, and their companions.

### Cohort study

Since the average incubation period of hepatitis A is 28 days, many people who visited the restaurant were expected to be in their incubation period. As it was necessary to identify onset and prevent the disease transmission, all visitors were included in the cohort study. Through interviews with the restaurateur, it was confirmed that most customers pay by credit card, so the KDCA was requested to secure the list of people who had paid by card at the restaurant from June 1 to its last day before suspension, July 28.

**Figure 1.**
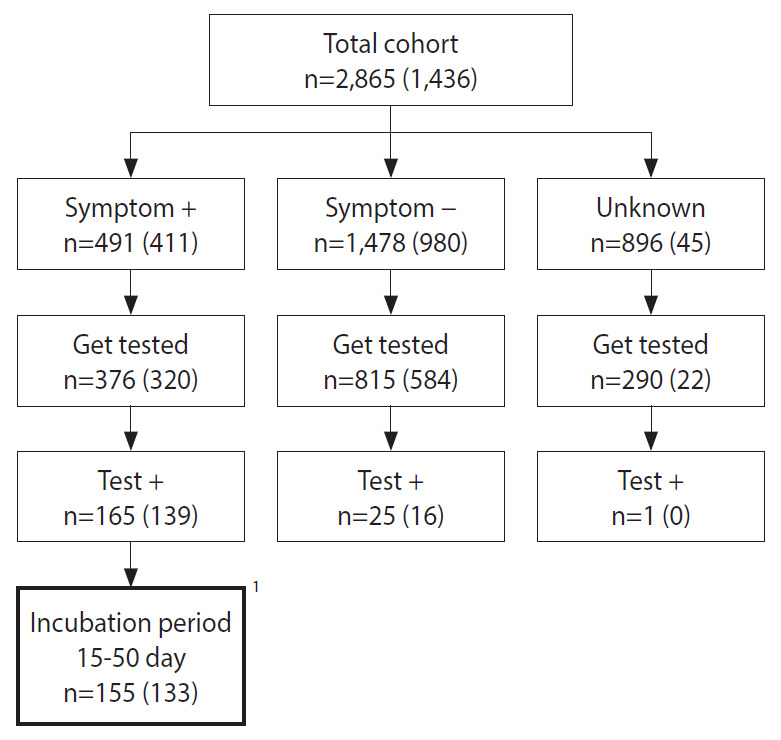
Study flow of the restaurant A visitor cohort study. The numbers in parentheses indicate the number of people who responded to the food intake history. ^1^Eligible for case definition.

During the investigation period, there were 1,149 people had paid by card at the restaurant. These people were given calls so that they could provide the names and contacts of 1,716 companions. For the entire the cohort of 2,865 people, it was checked whether they had visited the restaurant during the exposure period, followed by standardized questionnaires that surveyed the presence of hepatitis A-related symptoms and whether people had consumed certain food items provided at the restaurant. Among the total cohort, 2,431 (84.9%) people responded to at least a part of the telephone survey. There were 19 food items provided at the restaurant and there were 1,436 (50.1%) people who had responded to the survey items querying whether they had ingested certain food items at the restaurant. A total of 1,558 (54.4%) participants visited public health centers. Specimen collection was recommended for everyone regardless of the presence of symptoms, and 1,481 (51.7%) people agreed to undergo specimen collection: blood sample, stool, or rectal swab (Figure 1). Specimens were sent to the Busan Institute of Health and Environment for testing. Serum IgM anti-HAV antibodies test or RT-PCR test for hepatitis A along with genotype analysis of the HAV were conducted.

### Statistical analysis

Among 155 patients who met the case definition, excluding two patients whose date of symptom onset could not be confirmed, 153 patients were used to describe the weekly epidemic curves. From the 155 patients, 29 were excluded since they had visited the restaurant two or more times, and two were excluded because they could not remember when they had visited the restaurant. For the remaining 124 patients, the distribution of dates of their restaurant visits has been presented on a weekly basis.

To calculate the bivariate relative risk (RR) for specific food items, an analysis has been conducted using data from 1,436 people who have responded to the survey on food item ingested. For each food item, responses that had been left blank or those saying “I don’t know” have been excluded from the analysis. Multivariate logistic regression analysis was conducted using the responses of 1,321 people, excluding 115 people that had not responded survey questionnaires in one or more of the key variables, such as sex and age. Considering the fact that exposure to multiple food items leads to high correlations between the variables, the final model was selected in three steps. First, the base model that includes demographical variables and date of exposure is determined (step 1); then, the most appropriate model for each category — the main dish, the side dish, meals and beverages, and sauces — was selected using the Akaike information criterion (AIC) (step 2). Following the analysis for each food item category, all possible combinations created from the models selected for the food item categories were analyzed, from which the most appropriate model was selected using the AIC (step 3). The analysis was conducted using Stata version 16.0 (StataCorp., College Station, TX, USA).

### Ethics statement

This study is the results of an epidemiological investigation conducted by the national and local health authority directly in accordance with the law in a situation that requires urgent action for public health.

## RESULTS

### Descriptive epidemiology

From a cohort of 2,865, 191 had positive results on the laboratory tests. Moreover, according to the case definition, 26 asymptomatic people and 10 people that experienced the onset of symptoms outside the 15-50 days incubation period following their visit to the restaurant, have been excluded from the analysis (Figure 1). Therefore, 155 patients met the case definition, and the attack rate (AR) was 5.4%. There were 150 cases in Busan and five cases in the neighboring provinces. In district B, where the restaurant is located, there were 51 cases. The AR by age, calculated using available information, were 2.9% (2 of 68) for age < 20 years, 6.8% (38 of 555) for people in their 20s, 8.2% (73 of 886) for people in their 30s, 6.9% (34 of 495) for people in their 40s, 2.1% (6 of 283) for people in their 50s, and 0.3% (2 of 578) for people whose age was unknown. The ARs by sex were 8.8% (100 of 1,137) for male and 5.6% (52 of 924) for female (Table 1).

Among the related symptoms of hepatitis A, fever was the most common at 63.2% (98 of 155). Fatigue was reported in 80 patients (51.6%), jaundice in 65 patients (41.9%), abdominal pain in 60 patients (38.7%), and vomiting in 60 patients (38.7%).

**Figure 2.**
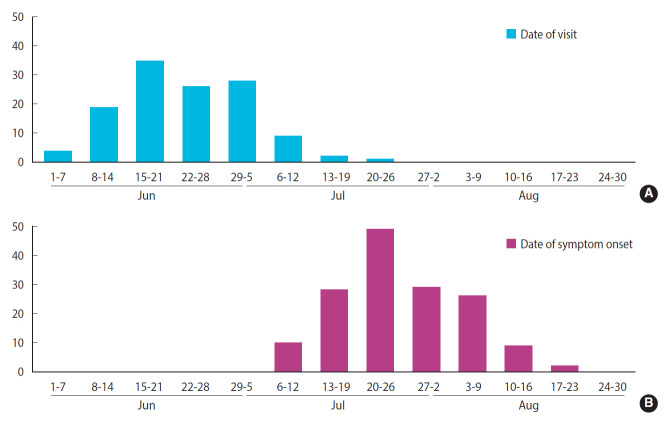
Distribution of date of visit (A) and epidemic curve (B).

The distribution of patients’ date of visit to the restaurant and epidemic curve are presented in Figure 2. Their dates of visit ranged from June 3 to July 24. Moreover, 108 patients (87.1%) had visited the restaurant during a period of 4 weeks, between June 8 and July 5. The date of symptom onset was distributed between July 7 and August 17 in the form of a unimodal epidemic curve, with the fourth week of July (July 20 to 26) as its peak. For values of the period between restaurant visit and date of symptom onset, the median was 31 days, the minimum was 16 days, and the maximum was 50 days.

In 90 patients, the genotype of their HAV was analyzed: 89 were type IA, and one was type IB.

### Relative risk of consuming each food item

Table 2 lists the HAV ARs and the RRs in those who consumed compared to those who have not consumed each food item. The group that had ingested salted clams had an AR of 17.3%, while the group that had not ingested salted clams had an AR of 0.2%: the RR was highest for salted clams at 96.12 (95% confidence interval [CI], 13.48 to 685.40). Groups with statistically significant RRs were those what consumed sliced pork at 1.63 (95% CI, 1.11 to 2.39), green onion kimchi at 2.43 (95% CI, 1.00 to 5.86), leaf mustard kimchi at 3.11 (95% CI, 1.28 to 7.55), seasoned soybean paste at 2.60 (95% CI, 1.08 to 6.29), salted shrimp at 2.05 (95% CI, 1.38 to 3.04), salted guts of hairtail at 2.20 (95% CI, 1.47 to 3.28), and kimchi stew at 1.58 (95% CI, 1.03 to 2.43).

Model selection process was presented in Supplementary Material 1. The AR for female was 0.49 times (95% CI, 0.30 to 0.80) the AR for male (Table 3). By periods, the week of June 15-21 had 9.24 times (95% CI, 3.34 to 25.55) higher compared to the week of June 1-7, followed in order by the week of June 29-July 5 at 4.74 times (95% CI, 1.71 to 13.09) and week of June 22-28 at 3.40 times (95% CI, 1.20 to 9.63). As for food ingestion, the development of hepatitis A was 68.62 times (95% CI, 9.22 to 510.87) more likely for those who consumed salted clams and 1.62 times (95% CI, 1.01 to 2.60) more likely for those who consumed sliced pork. For plain rice, the OR indicated a negative correlation of 0.52 times (95% CI, 0.32 to 0.84), and the ingestion of seasoned soybean paste was correlated with “I don’t know” responses, being 7.92 times (95% CI, 1.31 to 47.89) more likely. In summary, ingestion of salted clams and sliced pork between June 8 and July 5 posed the higher risk of being infected with hepatitis A.

### Control measures

Patients with hepatitis A were educated on how to prevent further transmission of the virus, and vaccines were administered to family members. During the investigation period, there were five people working at the restaurant including the restaurateur, and three of them were diagnosed with hepatitis A on July 29: they reported that they had consumed salted clams. For the three people diagnosed with hepatitis A, they were not allowed to return to work until their symptoms were completely relieved. For those who had visited the restaurant between June 1 and July 28, they were offered free diagnostic tests and vaccines for hepatitis A following the exposure: 984 people were administered vaccine shots. Moreover, they were asked to report immediately to public health centers in case they develop symptoms within the maximum incubation period of 50 days.

### Traceback investigation

On July 22, a field investigation was conducted at the restaurant, and evidence that could provide information on the purchase channel of food supplies, such as receipts, was obtained. Samples were collected from salted shrimp, salted guts of hairtail, green onion kimchi, leaf mustard kimchi, kitchen knives, and cutting boards that were being kept at the restaurant. However, HAV was not detected in these samples. Salted clams were not found the field investigation on July 22. However, during the second field investigation on August 8, an opened container of salted clams was found where the HAV genotype 1A was later detected on August 19. The salted clams provided at the restaurant were manufactured by a company located in Chungnam Province, which was elaborated by purchase receipts: on July 24, the local public health center was notified of this fact and were asked to conduct investigations on the company. In an unopened package of salted clams found at the company, HAV genotype 1A was detected, and the city of Busan was notified of this fact on August 28. The city of Busan sent requests to the Ministry of Food and Drug Safety for follow-up measures. From September 11 to September 25, the Ministry of Food and Drug Safety collected 136 different products of salted clams that were being distributed in Korea to run HAV tests on them. As a result, the HAV was detected in 44 (30 domestic, 14 imported from China) of the salted clam products, and the Ministry of Food and Drug Safety advised people not to eat salted clams on September 27 through an official recommendation [13].

## DISCUSSION

The investigation on this outbreak was conducted as a cohort study, using information obtained through an inquiry for the list of customers who paid by card over a 2-month period. The current study is significant as this was the first foodborne infectious disease epidemiological investigation in Korea using a list of customers who paid by card to establish the total cohort, which was then used to conduct the actual study.

The source of infection for the hepatitis A outbreak that occurred at a restaurant in Busan was determined to be salted clams. The distribution of incubation period had a median of 31 days, which was identical to the average incubation period of hepatitis A, and the results of a multivariate logistic regression analysis indicated that the ingestion of salted clams increased the risk of hepatitis A by 68.12 times (95% CI, 9.22 to 510.87). Moreover, the genotype analysis of specimens collected from 90 patients indicated that 89 of them had HAV genotype IA, which was identical to the genotype of the HAV detected in the salted clams found in an unopened container that was secured during the traceback investigation. The ingestion of semi-cooked or uncooked filter feeding bivalve shellfish is a common cause of outbreaks involving gastrointestinal infections transmitted via the fecal–oral route, such as the norovirus or hepatitis A [14,15]. Since the viruses excreted by patients with hepatitis A cannot be removed sufficiently through sewage treatment [16], they end up contaminating coastal waters: the habitat of filter-feeding bivalve shellfish. Due to the filter-feeding nature of the bivalve shellfish, these viruses are easily concentrated in the digestive organs of the shellfish [14]. The main source of infection of the 1988 mass outbreak of hepatitis A in Shanghai, China, which led to 290,000 reported cases of hepatitis A, was determined to be uncooked clams [6].

For the current study, hepatitis A ARs by age were 2.9% for ages less than 20 years, 6.8% for people in their 20s, 8.2% for people in their 30s, 6.9% for people in their 40s, and 2.1% for people aged > 50 years, with people in their 20s, 30s, and 40s showing relatively high ARs. This coincides with the 2019 study showing that the hepatitis A IgG antibody positive rates in Korea were 61.7% for people in their 10s, 32.8% for people in their 20s, 32.4% for people in their 30s, 63.2% for people in their 40s, and ≥ 95% for people in their 50s [17,18]. The results of the multivariate analysis indicated that there were no statistically significant differences between groups of ARs by age. The effect of age seems to have disappeared due to the correlation between the strongest risk factor—ingestion of salted clams—and age. Moreover, the AR of female appeared to be lower in the bivariate and multivariate analyses. Given that the antibody positive rate of Korean adults does not show statistically significant differences by sex [18-20], this could be the result of salted clam consumption that was not included in the survey results or specific qualities of the exposed group that are different from the general population. Other food items that indicated statistically significant differences in AR—such as sliced pork, seasoned soybean paste, and plain rice—are thought to be the result of cross-contamination or actual or residual confounding caused by inter-item correlation. Since salted shrimps are in a selectional relationship with salted clams, it was thought to have indicated a negative correlation, although it was not statistically significant.

The average incubation period of hepatitis A is long, at 28 days (minimum 15 days, maximum 50 days) [21], so it is easily confused for a case of sporadic that stretches over a few months when low-level contaminations are sustained or small clusters occur simultaneously in multiple regions: it is also difficult to find the relationship between hepatitis A and the food item that serves as the source of infection [22]. For the hepatitis A epidemic in 2019 in Korea, although it had started in March, the fact that it had been caused by a single type of food item was not known until the Ministry of Food and Drug Safety recommended on September 27 to stop consumption of salted clams [13]. As a result, people were constantly exposed to salted clams for months. After people were recommended to avoid uncooked clams and food sampling test standards for salted clams were expanded, the number of hepatitis A decreased rapidly. However, the reality is that, even in 2020 and 2021, there are continuous reports of small-scale outbreaks of hepatitis A caused by salted clams [23,24]. To prevent people from ingesting uncooked clams, there needs to be more publicity efforts regarding the dangers of uncooked clams; the food sampling test standards for salted clams should also be expanded. Moreover, to recognize outbreaks early in their occurrence from reported cases of hepatitis A, the local health authority must commit efforts to monitor the spatial and temporal clustering of cases and conduct detailed interviews with patients. Furthermore, for hepatitis A outbreaks that are caused by food items that have complex and international supply chains, there have been multiple cases where the outbreak was recognized through molecular genetics analysis [7,25,26]. Therefore, to detect hepatitis A outbreaks early, Korea needs a surveillance system based on laboratory molecular genetics. For the current study, the identical genotype of HAV was detected in the human samples and salted clams, but there were no phylogenetic analysis results based on molecular genetics. Laboratory surveillance data must be accumulated, which can then be utilized to prove the causal relationship between outbreaks and food items that serve as the source of infection.

The current study has the following limitations. First, IgG antibody tests could not be conducted on the participants. Strictly speaking, the AR must be calculated using only the susceptibles, which can be conducted by excluding IgG-positive participants from the denominator. In future epidemiological investigations of hepatitis A, the IgG test must be included to derive accurate ARs and lead to accurate analysis of results. Furthermore, since only 376 (76.6%) of 491 symptomatic patients were tested (Figure 1), the AR could have been calculated to be lower than the actual rate. Second, the response rate for the food item ingestion survey was low at 50.1%, and the amount consumed was not recorded. Since the amount ingested can have a significant effect, this needs to be evaluated and can be an important factor for evaluating the causality via the analysis of dose-response relationship. Third, since the study created the list using only the customers who paid by card, people who paid by cash have been excluded from the cohort. According to the Bank of Korea, the proportion of people who paid by cash at restaurants and cafes in 2019 was 16.4%, which tended to be higher for people in older age groups [27]. To minimize the exclusion of people who paid by cash, there needs to be a legal basis for securing the list of people who paid by cash and asked for a cash receipt.

Since the IgG antibody-positive rate is low for age groups between 20s and 40s, the susceptible population is large in Korea, which leads to the occurrence of hepatitis A outbreaks every few years [10]. Moreover, since the severity of hepatitis A increases with age [21], the severity is expected to increase along with the age of the current susceptible population. To detect outbreaks early in their occurrence, surveillance systems based on molecular genetics must be established, and the epidemiological investigation capabilities of regional govermnents must be strengthened. Furthermore, there needs to be a more comprehensive testing for food items that could potentially be contaminated with the HAV.

## Figures and Tables

**Table 1.** Attack rate by age group, sex, and visit date

Variables	Total cohort	No. of cases	Attack rate (%)
Total	2,865	155	5.4
Age (yr)			
<20	68	2	2.9
20-29	555	38	6.8
30-39	886	73	8.2
40-49	495	34	6.9
≥50	283	6	2.1
Unknown	578	2	0.3
Sex			
Male	1,137	100	8.8
Female	924	52	5.6
Unknown	804	3	0.4
Visit date			
Jun 1-7	352	6	1.7
Jun 8-14	326	22	6.7
Jun 15-21	348	43	12.4
Jun 22-28	393	31	7.9
Jun 29-Jul 5	380	38	10.0
Jul 6-12	320	11	3.4
Jul 13-19	326	3	0.9
Jul 20-28	379	1	0.3
Unknown	41	0	0.0

**Table 2.** RR for ingestion of each food item

Food item	Attack rate (%)		RR (95% CI)
	Ingestion group^1^	Non-ingestion group^2^	
Pork belly	7.8 (98/1,256)	6.4 (7/109)	1.22 (0.58, 2.55)
Pork neck	8.9 (73/821)	6.3 (32/511)	1.42 (0.95, 2.12)
Sliced pork	9.7 (62/636)	6.0 (40/669)	1.63 (1.11, 2.39)
Green onion kimchi	8.3 (100/1,204)	3.4 (5/146)	2.43 (1.00, 5.86)
Leaf Mustard kimchi	8.3 (96/1,153)	2.7 (5/187)	3.11 (1.28, 7.55)
Seasoned soybean paste	8.2 (99/1,203)	3.2 (5/158)	2.60 (1.08, 6.29)
Salted shrimp	10.9 (64/587)	5.3 (36/677)	2.05 (1.38, 3.04)
Salted clams	17.3 (130/752)	0.2 (1/556)	96.12 (13.48, 685.40)
Salted guts of hairtail	11.1 (68/611)	5.1 (33/652)	2.20 (1.47, 3.28)
Sesame oil with salt	7.6 (74/978)	8.0 (27/339)	0.95 (0.62, 1.45)
Pickled vegetables	7.6 (71/935)	8.4 (29/346)	0.91 (0.60, 1.37)
Lettuce	7.4 (77/1,044)	8.8 (27/306)	0.84 (0.55, 1.27)
Sesame leaf	7.7 (80/1,038)	7.8 (24/308)	0.99 (0.64, 1.53)
Pepper	7.7 (74/955)	7.1 (28/397)	1.10 (0.72, 1.67)
Garlic	7.2 (76/1,057)	8.9 (26/292)	0.81 (0.53, 1.24)
Boiled rice	6.7 (61/913)	9.9 (42/425)	0.68 (0.46, 0.98)
Kimchi stew	8.7 (72/824)	5.5 (27/489)	1.58 (1.03, 2.43)
Soybean paste stew	5.9 (21/357)	8.3 (79/948)	0.71 (0.44, 1.12)
Spicy noodles	6.7 (6/90)	7.9 (98/1,247)	0.85 (0.38, 1.88)
Water	7.5 (90/1,205)	9.6 (14/146)	0.78 (0.46, 1.33)

RR, relative risk; CI, confidence interval.

1 Number of cases/number of people who ingested the food item.

2 Number of cases/number of people who did not ingest the food item.

**Table 3.** Association between food consumed and hepatitis A cases

Variables	aOR (95% CI)
Age (yr)	
0-29	1.00 (reference)
30-39	1.53 (0.86, 2.70)
40-49	1.05 (0.54, 2.04)
≥50	0.37 (0.11, 1.18)
Sex	
Male	1.00 (reference)
Female	0.49 (0.30, 0.80)^***^
Visit date	
Jun 1-7	1.00 (reference)
Jun 8-14	2.77 (0.94, 8.18)
Jun 15-21	9.24 (3.34, 25.55)^***^
Jun 22-28	3.40 (1.20, 9.63)^**^
Jun 29-Jul 5	4.74 (1.71, 13.09)^***^
Jul 6-12	1.29 (0.32, 5.13)
Jul 13-19	0.38 (0.04, 3.42)
Jul 20-28	0.45 (0.05, 4.12)
Seasoned soybean paste	
No	1.00 (reference)
Unknown	7.92 (1.31, 47.89)^**^
Yes	2.55 (0.93, 7.02)
Salted shrimp	
No	1.00 (reference)
Unknown	0.70 (0.22, 2.21)
Yes	0.70 (0.43, 1.14)
Salted clams	
No	1.00 (reference)
Unknown	5.10 (0.27, 96.43)
Yes	68.62 (9.22, 510.87)^***^
Sliced pork	
No	1.00 (reference)
Unknown	1.43 (0.48, 4.31)
Yes	1.62 (1.01, 2.60)^**^
Boiled rice	
No	1.00 (reference)
Unknown	0.41 (0.12, 1.42)
Yes	0.52 (0.32, 0.84)^***^

aOR, adjusted odds ratio; CI, confidence interval.

** p<0.01,

*** p<0.001.

## SUPPLEMENTARY MATERIALS

Supplementary Material 1. Model selection process

Korean version is available at https://www.e-epih.org/.
