# Supplementary material for: An outbreak of hepatitis A associated with salted clams in Busan, Korea
Source: Epidemiol Health. 2021 Dec 29;44:e2022003. doi: 10.4178/epih.e2022003 (PMC8989951; doi:10.4178/epih.e2022003)
Supplement: Supplementary Material 1. — Model selection process [file epih-44-e2022003-suppl1.docx]

Supplementary Material 1. Model selection process

|  |  |  | Variables included | AIC |
| --- | --- | --- | --- | --- |
| Step 1 | General Characteristics | M0 |  | 1207.7 |
|  |  | M1 | Age | 1115.3 |
|  |  | M2 | Gender | 1081.4 |
|  |  | M3 | Age gender | 1034.2 |
|  |  | M4 | Age gender visit week | 944.5 |
| Step 2 | Main Dish | M5 | M4 + Pork belly(MD1) | 649.8 |
|  |  | M6 | M4 + Pork neck(MD2) | 646.7 |
|  |  | M7 | M4 + Sliced pork(MD3) | 644.7 |
|  | Side dish | M8 | M4 + Green onion kimchi(SD1) | 647.4 |
|  |  | M9 | M4 + Leaf Mustard Kimchi(SD2) | 644.5 |
|  |  | M10 | M4 + Pickled vegetables(SD3) | 649.3 |
|  |  | M11 | M4 + Lettuce(SD4) | 650.0 |
|  |  | M12 | M4 + Sesame leaf(SD5) | 650.0 |
|  |  | M13 | M4 + Pepper(SD6) | 650.1 |
|  |  | M14 | M4 + Garlic(SD7) | 648.8 |
|  | Meal  & Water | M15 | M4 + Boiled rice (M1) | 645.8 |
|  |  | M16 | M4 + Kimchi stew (M2) | 648.1 |
|  |  | M17 | M4 + Soybean paste stew (M3) | 649.2 |
|  |  | M18 | M4 + Spicy noodles (M4) | 649.7 |
|  |  | M19 | M4 + Water(W) | 649.9 |
|  | Sauce | M20 | M4 + Seasoned soybean paste(S1) | 644.4 |
|  |  | M21 | M4 + Salted shrimp(S2) | 636.2 |
|  |  | M22 | M4 + Salted clams(S3) | 672.7 |
|  |  | M23 | M4 + Salted guts of hairtail(S4) | 641.5 |
|  |  | M24 | M4 + Sesame oil with salt(S5) | 649.6 |
|  |  | M25† | M4 + S1+S2+S3 | 567.2 |
| Step 3 | Final Model | MF1 | M4 + S1+S2+S3 | 567.2 |
|  |  | MF2 | M4 + S1+S2+S3+MD3+SD2+M1 | 565.0 |
|  |  | MF3 | M4 + S1+S2+S3+MD3+SD2 | 569.0 |
|  |  | MF4 | M4 + S1+S2+S3+MD3+M1 | 563.7 |
|  |  | MF5 | M4 + S1+S2+S3+SD2+M1 | 564.5 |
|  |  | MF6 | M4 + S1+S2+S3+MD3+SD2 | 567.3 |
|  |  | MF7 | M4 + S1+S2+S3+SD2 | 568.6 |
|  |  | MF8 | M4 + S1+S2+S3+MD3 | 563.8 |
|  |  | MF9 | M4 + S1+S2+S3+M1 | 567.2 |

†Results of possible combinations of the five sauces are not presented. Only the results of the most suitable combinations are presented.

AIC, Akaike information criterion
