# Supplementary material for: An outbreak of hepatitis A associated with salted clams in Busan, Korea
Source: Epidemiol Health. 2021 Dec 29;44:e2022003. doi: 10.4178/epih.e2022003 (PMC8989951; doi:10.4178/epih.e2022003)
Supplement: Supplementary file 2 [file epih-44-e2022003-suppl2.docx]

**Title**

An outbreak of hepatitis A associated with salted clams in Busan, South Korea

**Abstract**

Background: 2019년 7월, 부산시의 한 식당을 방문한 사람에서 다수의 A형간염 환자가 신고되었다. 감염원 규명과 확산 방지를 위해 실시한 역학조사 결과를 제시하고 A형간염 관리를 위해 보완할 점을 제시하고자 하였다.

Methods: 2019년 6월 1일부터 7월 28일까지 A 식당을 방문하여 카드 결제를 한 사람과 동반자 2,865명 전체를 대상으로 코호트 조사를 수행하였다. 표준화된 설문지를 이용하여 A형간염 증상 여부와 19개 음식의 섭취여부를 조사하였으며, 이 중 보건소를 방문한 사람을 대상으로 검체를 채취하고 백신 접종을 하였다.

Results: A형간염에 확진 된 사람은 총 155명으로 발병률은 5.4%였다. 7월 넷째 주를 정점으로 하는 단봉형 유행곡선을 보였으며 식당 방문일에서 증상 발생일까지 기간의 중앙값은 31일이었다. 환자 90명의 유전형 분석 결과 1A형이 89명이었다. 식당에서 제공된 음식 중 조개젓(Salted clams) 섭취군의 발병률은 17.3%였고 비섭취군의 발병률은 0.2%로 상대위험도가 96.12 (95% CI, 13.48 to 685.40)로 가장 높았다. 다변량 로지스틱 회귀분석 결과 조개젓 섭취는 A형간염 발병 위험을 68.62배(95% CI, 9.22 to 510.87) 높였다. 역추적 조사를 통해 확보한 미개봉 조개젓에서 1A 유전형 A형간염 바이러스가 검출되었다.

Conclusions: 익히지 않은 조개류는 A형간염과 같이 분변-구강 경로로 전파되는 바이러스성 장관감염증의 집단발생을 일으키는 흔한 원인이다. 익히지 않은 조개류를 섭취하지 않도록 더욱 홍보를 강화해야 하며, 조개젓에 대한 식품 표본 검사를 더욱 확대할 필요가 있다. 또한 유행을 조기에 발견할 수 있도록 분자유전학적 감시체계를 확립하고 지역의 역학조사 역량을 강화해야 한다.

Keywords: Hepatitis A, salted clams, food-borne outbreak, South Korea

**Introduction**

A형간염 바이러스는 *Picornaviridae* 에 속한 RNA virus로 사람이 유일한 자연 숙주이다 [1]. 중앙 및 남아메리카, 아프리카, 중동, 아시아, 서태평양 지역이 A형간염 고풍토 지역으로 알려져 있다. 발열, 무력감, 식욕 감퇴, 복통, 구토, 황달이 A형간염의 주요 증상이며 6세 미만 소아의 경우 70%가 무증상이고 연령이 증가할수록 증상 발현이 많아져 50세 이상 연령군에서는 치명률이 1.8%에 이른다. 1991년 유럽에서 처음 백신이 허가되었으며 2회 접종으로 95% 이상의 효과를 보인다. A형간염 백신은 노출 후 예방 목적으로 접종할 수 있으며 노출 후 2주 이내에 접종하는 경우 매우 높은 예방 효과를 보인다 [2].

A형간염 바이러스는 사람 간 접촉이나 오염된 물 또는 음식을 통해 분변-구강 경로로 전파된다. A형간염 발생이 매우 낮은 국가에서도 다양한 상황과 음식을 통해 A형간염 유행이 보고되고 있다. 병원이나 요양원에서 중환자나 노인 또는 소아를 돌보는 경우 [3,4], 노숙인과 같이 위생상태가 좋지 않은 상황 [5]에서 A형간염 유행이 일어날 수 있다. 음식의 경우 섭취 전 가열하지 않거나 충분히 가열하지 않고 먹는 재료인 조개[6], 냉동과일[7], 반건조 과일[8], 야채[9] 등의 재료를 통한 유행이 보고되었다.

한국에서는 2011년 A형간염이 전수 감시 감염병으로 지정되었다. 2011년부터 2018년까지 가장 많은 환자가 신고된 해는 2011년 5,521명이었고, 가장 적은 환자가 신고된 해는 2013년 867명이었다 [10]. 2019년에는 17,598명의 환자가 신고되었는데, 질병관리청에서는 2019년 6월 25일과 7월 26일 일부 소규모 집단 사례의 감염원을 조개젓으로 발표하였다 [11,12]. 그러나 2018년 동기간 대비 6배 이상 신고 환자가 증가한 원인이 충분히 설명되지는 못하고 있는 상황이었다.

2019년 봄부터 대전, 충청, 수도권을 중심으로 A형간염 환자가 큰 폭으로 증가하고 있었으나 부산의 경우 7월 초까지는 주간 A형간염 환자 신고 수가 5건 내외로 예년과 비슷한 수준이었다. 그러던 중 2019년 7월 둘째 주(7월 6일 – 7월 12일)에 9명, 셋째 주(7월 13일 – 7월19일)에 18명의 A형간염 환자가 신고되어 예년에 비해 A형간염 환자 신고가 증가하였다는 것을 인지하였다. 7월 둘째 주와 셋째 주에 신고된 환자의 공간적 집적성을 확인한 결과 부산시의 16개 구 중 B 구에서 8명의 환자가 신고되었음을 확인하였다. 주말 이후 7월 22일 월요일 하루에만 15명의 A형간염 환자가 신고되어 15명을 대상으로 전화 또는 대면 심층 인터뷰를 진행하였다. 그 결과 15명 중 12명이 6월 중 B 구에 위치한 A 식당을 방문한 것으로 확인되어 A 식당을 매개로 한 유행으로 판단하였다. 부산시와 감염병관리지원단은 감염원을 규명하고 확산을 방지하기 위해 7월 23일 역학조사팀을 꾸리고 본격적인 역학조사에 착수하였다.

**Methods**

**Case definition and case finding**

환자 정의는 2019년 6월 1일부터 2019년 7월 28일까지 A 식당을 방문한 사람 중 15-50일 이내에 발열, 무력감, 황달, 복통, 구토 등 A형간염에 합당한 하나 이상의 증상이 있으면서 Serum IgM anti-HAV antibodies 검사 양성 또는 A형간염 특이 유전자 검출을 위한 Reverse transcription polymerase chain reaction(RT-PCR) 검사에서 양성인 경우로 하였다.

2019년 6월 1일부터 7월 28일까지 A 식당의 카드 결제자와 동반자 전체를 대상으로 코호트를구축하고 발병 여부를 조사하였다.

**Cohort study**

A형간염의 평균 잠복기는 28일로 해당 식당들 방문한 사람 중 다수가 잠복기 상태에 있을 것으로 예상되었다. 따라서 식당 방문자 전체를 대상으로 발병 예방과 전파 차단 조치가 필요하여 방문자 전체를 대상으로 코호트 조사를 실시하기로 하였다. 조사 시작 시점에서 A식당 방문력이 확인된 환자들의 식당 방문일이 6월 1일 이후였으므로 조사 대상자는 6월 1일 이후 방문자로 하였고 조사를 진행하면서 6월 1일 이전 방문자 중 A형간염 환자가 발견되는 경우 조사 기간을 확대하기로 하였으나 6월 1일 이전 방문자 중 환자 보고는 없었다. 업주와의 면담을 통해 식당 방문자 대부분이 카드결제를 한다는 사실을 확인하고, 질병관리청에 6월 1일부터 A 식당이 영업을 한 마지막 날인 7월 28일까지 카드결제자 명단 확보를 요청하였다. 카드결제자 명단에는 카드 소유주의 이름과 생년월일, 결제일, 전화번호가 포함되도록 요청하였다.

해당 기간 동안 A 식당 카드 결제자는 총 1,149명이었다. 이들에게 전화를 걸어 같이 식당을 방문하여 식사 한 동반자 1,716명의 이름과 연락처를 조사하였다. 해당기간 방문자 전체 코호트 총 2,865명을 대상으로 방문력을 다시 확인하고 A형간염 관련 증상 여부와 음식 별 섭취여부를 표준화된 질문지를 통해 전화 조사하였다. 전화조사는 부산시 내 16개 보건소의 조사 지침 교육을 받은 사람이 수행하였다. 전체 코호트 중 전화 조사에 일부라도 응답한 사람은 2,431명(84.9%)이었다. A 식당에서 제공된 음식은 반찬을 포함하여 총 19가지였으며 음식 별 섭취여부에 응답한 대상자는 총 1,436명(50.1%)이었다. 전화 조사에 응한 사람 중 1,558명(54.4%)은 보건소를 방문하였다. 증상 여부에 관계없이 검체 채취를 권고하였고 이중 1,481명(51.7%)은 혈액, 대변 또는 직장도말 검체 채취에 응하였다 (figure 1). 검체는 부산시 보건환경연구원에 검사 의뢰하였다. Serum IgM anti-HAV antibodies 검사 또는 A형간염 특이 유전자 검출을 위한 RT-PCR 검사와 A형간염 바이러스 유전형 분석이 진행되었다. 코호트 조사는 8월 10일부터 9월 5일까지 진행되었다.

**Data analysis**

환자 정의에 부합하는 155명 중 증상발생일이 확인되지 않은 2명을 제외한 153명의 증상발생일을 기준으로 주별 유행곡선을 기술하였다. 155명 중 A식당을 2회 이상 방문한 29명과 방문일을 기억하지 못한 2명을 제외한 124명의 식당 방문일 분포를 주별로 제시하였다. 124명의 식당 방문일부터 증상 발생일까지 기간을 중앙값, 최솟값, 최댓값을 제시하였다.

음식 별 이변량 비교위험도 산출을 위해 식품 섭취력에 응답한 1,436명을 대상으로 분석을 진행하였고, 각 음식 별로 모름 또는 무응답인 경우는 제외하였다. 다변량 로지스틱 회귀분석에는 성별과 연령 등 주요 변수의 정보가 없는 115명을 제외하고 1,321명을 분석 대상으로 하였다. 식품섭취 여부 조사 항목에서 모름이 표기될 경우 전체 사례에서 제외되게 되어 이들로 인한 바이어스 가능성을 최소화하기 위해 모름을 별도의 분류로 포함하여 가능한 모든 조사대상자에 대한 분석을 시행하였다. 다수의 음식에 노출되어 변수간 상관성이 높음을 고려하여 서로의 효과를 고려하여 살펴볼 수 있는 다변량 분석을 시행하기 위해서 3단계로 최종 모형을 선택하였다. 우선 인구학적 변수 및 노출일을 포함한 기본 모형을 결정하고(Step 1), 이 기본모형에 주 요리(Main dish), 반찬(Side dish), 식사와 음료, 소스별로 Akaike information criterion(AIC)를 기준으로 가장 적합한 모형을 산출하였다. 그런데, 소스는 동시에 여러 개를 섭취할 수 있어 5개 소스의 모든 조합을 대상으로 추가 분석을 시행하여 가장 적합한 조합을 선택하였다(Step 2). 주요 음식군 별 분석이후 주 요리, 반찬, 식사와 음료, 소스 별 가장 적합한 모형들을 대상으로 가능한 모든 조합에 대하여 분석을 시행하고 Akaike information criterion(AIC)를 기준으로 가장 적합한 모형을 선택하였다(Step 3). 최종 모형에 포함된 변수와 A형 간염 발생과의 관련성은 Adjusted Odds Ratio와 95% 신뢰구간을 제시하고 평가하였다. 분석은 Microsoft Excel 365 (Microsoft Corp., Redmond, WA, USA)와 STATA version 16.0 (Stata Corp., College Station, TX, USA)을 이용하였다.

**Results**

**Descriptive epidemiology**

전체 코호트 2,865 명 중 실험실 검사 결과 양성인 사람은 총 191명이었다. 이 중 증상이 없어 A 식당 방문일 이후 잠복기 내 발병 여부를 확인할 수 없었던 26명과 식당 방문일로부터 15-50일 간의 잠복기 내 증상이 발생하지 않은 10명은 환자 정의에 따라 제외하였다(figure 1). 따라서 환자 정의에 부합한 사례는 총 155명이었으며 발병률은 5.4%였다. 부산시에서 150명이 발생하였고 인접 province에서 5명이 발생하였다. A 식당이 위치한 B 구에서는 51명이 발생하였다. 전체 코호트 2,865 명 중 578명의 연령과 804명의 성별이 조사되지 않아 성별, 연령별 발병률을 정확히 산출할 수 없었다. 가용한 정보로 산출한 연령별 발병률은 20세 미만은 2.9%(68명 중 2명), 20대는 6.8%(555명 중 38명), 30대는 8.2%(886명 중 73명), 40대는 6.9%(495명 중 34명), 50대 이상은 2.1%(283명 중 6명), 연령 미상인 경우는 0.3%(578명 중 2명) 였다. 성별 발병률은 남성은 8.8%(1,137명 중 100명) 여성은 5.6%(924명 중 52명)였다(table 1).

A형간염 관련 증상 중 발열은 155명 중 98명(63.2%)에서 나타나 가장 빈도가 높았다. 무력감은 80명(51.6%), 황달은 65명(41.9%), 복통은 60명(38.7%), 구토는 60명(38.7%) 순이었다.

환자들의 A 식당 방문 날짜의 분포와 유행 곡선은 figure 2와 같다. A 식당을 한 번만 방문한 환자는 124명이었고, 방문일은 6월 3일에서 7월 24일까지 분포하였다. 6월 8일에서 7월 5일까지 4주 간 방문한 환자가 108명(87.1%)을 차지하였다. 증상 시작일은 7월 7일에서 8월 17일까지 분포하였으며, 7월 넷째 주(7월 20일 – 7월 26일)를 정점으로 하는 단봉형 유행곡선의 모습을 보였다. 식당 방문일로부터 증상 발생일까지 걸린 시간의 중앙값은 31일이었으며, 최소 16일, 최대 50일이었다.

환자 중 90명의 A형간염 바이러스 유전형이 분석되었으며, 89명은 IA형, 1명은 IB형이었다.

**Relative risk of consuming each food item**

A 식당에서 제공된 음식의 종류는 19 종류였다. 각 음식 별 섭취여부를 묻는 설문에 응답한 사람은 1,436명이었다. 사례정의에 부합한 155명 중에는 133명이 식품 섭취력 설문에 응답하였다. 각 음식별 섭취군과 비섭취군의 발병률과 상대위험도는 table 2와 같다. 조개젓(Salted clams) 섭취군의 발병률은 17.3%였고 비섭취군의 발병률은 0.2%로 상대위험도가 96.12 (95% CI, 13.48 to 685.40)로 가장 높았다. 대패 다리살(Sliced pork)을 먹은 그룹의 상대위험도는 1.63 (95% CI, 1.11 to 2.39), 파김치(green onion kimchi)는 2.43 (95% CI, 1.00 to 5.86), 갓김치(leaf mustard kimchi)는 3.11 (95% CI, 1.28 to 7.55), 쌈장(seasoned soybean paste)은 2.6 (95% CI, 1.08 to 6.29), 새우젓(salted shrimp)은 2.05 (95% CI, 1.38 to 3.04, 갈치속젓(salted guts of hairtail)은 2.2 (95% CI, 1.47 to 3.28), 김치찌개(kimchi stew)는 1.58 (95% CI, 1.03 to 2.43)로 유의하게 높았다.

관련요인을 함께 고려한 최종모형을 선택하기 위해 3단계로 진행한 모형선택과정에서 1단계의 기본 모형은 연령군, 성별, 방문 주 모두를 포함한 경우가 가장 적합한 모형이었다. 2단계의 주 요리(Main dish), 반찬(Side dish), 식사와 음료, 소스별 최적 모형 결과는 기본 모형의 포함변수와 함께 주 요리는 대패 다리살, 반찬은 갓김치, 식사의 경우 쌀밥, 소스의 경우는 쌈장, 새우젓, 조개젓 모두를 포함한 모형이었다. 주요 음식 별 결과를 활용한 최종 모형에는 쌈장, 새우젓, 조개젓, 대패 다리살, 쌀밥이 최종 선택되었다(supplementary table 1). 인구사회학적 변수와 노출 변수들과의 관련성에서는 여성의 발병률이 남성보다 0.49배(95% CI, 0.30 to 0.80)로 낮았다(table 3). 기간별로는 6/1~6/7일 주에 비해 6/15~6/21일 기간의 경우 9.24배(95% CI, 3.34 to 25.55)로 가장 높은 관련성을 보였고, 다음이 6/29~7/5일 기간이 4.74배(95% CI, 1.71 to 13.09), 6/22~6/28일 기간이 3.40배(95% CI, 1.20 to 9.63)의 순으로 높았고, 해당 주의 이전과 이후의 기간에서는 통계적으로 유의한 수준의 관련성이 나타나지 않았다. 섭취한 음식과의 관련성에서는 조개젓을 섭취한 경우 A형간염 발병이 68.62배(95% CI, 9.22 to 510.87) 증가하는 것으로 나타났고, 대패 다리살의 경우는 1.62배(95% CI, 1.01 to 2.60) 증가하는 것으로 나타났다. 쌀밥의 경우 OR은 음의 관련성인 0.52배(95% CI, 0.32 to 0.84)로 나타났고 쌈장의 섭취는 모름의 응답과 7.92배(95% CI, 1.31 to 47.89) 관련성이 있는 것으로 나타났다. 요약하면 6/8일부터 7/5일까지 조개젓과 대패 다리살을 먹은 경우가 A형간염 발생 위험을 높인 것으로 나타났다.

**Control measures**

A형간염 환자에게는 전파예방 교육을 하였고, 환자의 가정 접촉자에게는 노출 후 백신 접종을 하였다. 해당 기간 A 식당에서 근무한 사람은 업주를 포함하여 5명이었으며 이들 중 3명이 조사과정에서 7월 29일 A형간염에 확진되었고 조개젓을 섭취한 것으로 확인되었다. 이들은 증상이 완전히 소실될 때까지 업무 종사를 제한하였다. 6월 1일에서 7월 28일까지 A 식당을 방문한 사람들의 경우 A형간염 검사와 노출 후 백신접종을 무료로 제공하였으며 984명이 백신 접종을 받았다. 또한 최대 잠복기인 50일 이내에 증상이 발생할 경우 즉시 보건소로 신고해 줄 것을 당부하였다.

**Traceback investigation**

7월 22일 A 식당 현장조사를 실시하고 식자재 구입 경로를 확인할 수 있는 영수증 등을 확보하였다. A 식당에서 보관 중이던 새우젓, 갈치속젓, 파김치, 갓김치와 칼, 도마 표면 도말 검체를 확보하여 부산시 보건환경연구원에 검사 의뢰하였으나 A형간염 바이러스는 검출되지 않았다. 조개젓은 7월 22일 현장조사에서는 발견되지 않았으나 이후 8월 8일 2차 현장 조사에서 개봉된 상태로 확보하였고 8월 19일 1A 유전형 A형간염 바이러스가 검출되었다. A 식당에서 제공된 조개젓은 충청남도에 위치한 업체에서 제조된 것으로 구입 영수증을 통해 확인하였으며 7월 24일 해당 지역 보건소에 이 사실을 통보하고 조사를 요청하였다. 해당 업체에서 미개봉 상태로 수거된 조개젓을 질병관리청에서 검사한 결과 1A 유전형 A형간염 바이러스가 검출되어 8월 28일 부산시로 통보되었다. 부산시는 식품의약품안전처에 후속 조치를 요청하였다. 이후 식품의약품안전처는 9월 11일에서 25일까지 국내 유통 중인 조개젓 136건을 수거하여 A형간염 바이러스 유전자 검사를 실시하였다. 그 결과 그 중 44건(국산 30건, 중국산 14건)의 제품에서 A형간염 바이러스 유전자가 검출되었다고 발표하였고, 9월 27일 조개젓을 먹지 말 것을 공식 권고하였다 [13].

**Discussion**

이 유행조사는 카드결제자 정보 조회를 통해 코호트 연구 디자인으로 수행되었다. 두 달 간의 식당 방문자 카드 결제자 정보조회를 요청하여 1,149명의 명단을 확보하였고, 결제자에게 전화를 걸어 동반한 사람 1,716명의 명단을 조사하였다. 우리나라의 식품매개 감염병 역학조사에서 카드결제자 명단을 이용하여 전체 코호트를 구축하고 조사를 수행한 최초의 사례라는 점에서 큰 의미가 있다.

이번 부산시 한 식당을 매개로 발생한 A형간염 집단발생의 감염원은 조개젓으로 확정할 수 있다. 식당 방문일에서 증상 발생일 까지의 잠복기의 분포가 중앙값 31일로 A형간염의 평균 잠복기와 같았으며 다변량 로지스틱 회귀분석에서 조개젓 섭취군의 발병 위험이 68.62배(95% CI, 9.22 to 510.87) 높음을 확인하였다. 또한 환자 90명에서 검출된 바이러스 유전형 분석 결과 89명은 IA형이었으며 역추적 조사 결과 미개봉 조개젓에서 동일한 유전형의 A형간염 바이러스가 검출된 것을 그 근거로 들 수 있다. 90명 중 1명 만이 IB 유전형으로 확인되어 해당 식당에서의 노출과는 관련이 없는 사례가 연구에 포함되었을 가능성은 매우 낮음을 확인할 수 있었다. 덜 익히거나 익히지 않은 filter feeding bivalve shellfish의 섭취는 노로바이러스나 A형간염과 같이 분변 구강 경로로 전파되는 바이러스성 장관감염증의 집단발생을 일으키는 흔한 원인이다 [14,15]. A형간염 환자에서 배출된 바이러스는 하수 처리를 통해서 충분히 제거되기 어려우므로 [16] 조개류가 서식하는 인근 해역을 오염시킨다. 그 결과 조개류의 filter-feeding nature로 인해 소화기관에 쉽게 농축된다 [14]. 1988년 중국 상하이에서 29만명의 A형간염 환자가 발생한 대규모의 유행의 주요 감염원은 익히지 않은 조개로 밝혀진 바 있다 [6].

이 조사에서 연령별 A형간염 발병률은 20세 미만은 2.9%, 20대는 6.8%, 30대는 8.2%, 40대는 6.9%, 50대 이상은 2.1%로 20대, 30대, 40대가 상대적으로 높은 발병률을 보였다. 이는 2019년 우리나라 인구의 A형간염 IgG 항체양성률이 10대는 61.7%, 20대는 32.8%, 30대는 32.4%, 40대는 63.2%, 50대 이상은 95%이상이었던 연구와 일치하는 결과이다 [17,18]. 다변량 분석 결과 연령별 발생률의 차이는 없었다. 가장 강력한 위험요인인 조개젓 섭취와 연령 간 관련성으로 인해 연령 효과가 사라진 것으로 보인다. 또한 이변량 분석과 다변량 분석에서 여성의 발병률이 낮게 나타났다. 이는 우리나라 성인의 성별 항체양성률이 유의미한 차이가 없음을 감안하면 [18–20] 조사에 포함되지 않은 조개젓 섭취량 또는 일반 인구집단과는 다른 노출집단의 특이성으로 인한 결과일 수 있다. 다변량 분석에서 유의미한 발병률의 차이를 보인 음식인 대패 다리살, 쌈장, 쌀밥 등은 교차오염 또는 식품 간 상관성으로 인한 실제 또는 잔여 교란(residual confounding)의 가능성으로 생각된다. 새우젓의 경우 조개젓과 선택관계에 있으므로 통계적으로 유의하지 않은 음의 상관성을 보인 것으로 생각된다.

A형간염은 평균 잠복기가 28일 (최소 15일, 최대 50일)로 길어 [21] 낮은 수준의 유행이 지속되거나 여러 지역에서 유행하는 경우 수주에서 수개월 간의 산발 사례로 인지되기 쉽고 감염원이 되는 식품과의 연관성을 발견하기 어렵다 [22]. 2019년 우리나라 A형간염 유행의 경우도 3월 부터 시작된 유행이 9월 27일 식약처의 조개젓 섭취 중단 권고가 있을 때까지 수개월간 단일 식품에 의한 유행임을 알지 못하였다 [13]. 그 결과 원인 식품인 조개젓에 대한 노출이 수개월간 지속되는 결과를 낳았다. 익히지 않은 조개류를 섭취하지 않도록 권고하고 조개젓에 대한 식품 표본 검사가 확대된 이후 A형간염 환자 수는 급격하게 감소하였다. 그러나 2020년과 2021년에도 조개젓으로 인한 소규모 유행이 지속적으로 보고되고 있는 실정이다 [23,24]. 익히지 않은 조개류를 섭취하지 않도록 더욱 홍보를 강화해야 하며, 조개젓에 대한 식품 표본 검사를 더욱 확대할 필요가 있다. 또한 A형간염 신고 자료로 유행을 조기에 인지하기 위해서는 지역사회에서 시간적, 공간적 집적성이 있는지 여부를 지속적으로 살피고 환자를 대상으로 면밀한 인터뷰를 시행할 필요가 있다. 또한 국제적이고 복잡한 유통망을 가진 식품을 매개로 한 A형간염 유행의 경우 분자유전학적 분석을 통해 유행이 인지되고 이후 역학 조사를 통해 원인 식품을 밝혀낸 경우가 많다 [7,25,26]. 따라서 A형간염 유행을 조기에 인지하기 위해서는 우리나라에서도 분자유전학적 실험실 감시가 반드시 필요하다. 이번 조사에서도 인체 검체와 조개젓에서 동일한 유전형의 A형간염 바이러스가 검출되었으나 분자유전학적 계통 분석 결과는 제시하지 못하였다. 분자유전학적 실험실 감시 데이터를 축적하여 원인 식품과의 인과관계 증명에도 활용할 필요가 있다.

이 연구는 다음과 같은 제한점을 가지고 있다. 첫째, 연구 대상자의 IgG 항체 검사를 수행하지 못하였다. 엄밀한 의미에서의 발병률은 IgG 항체양성자를 분모에서 제외하여 감수성자만을 대상으로 해야 하며 이는 분석 결과의 해석에도 영향을 주는 요인이다. 향후 A형간염 역학조사에서는 IgG 검사를 포함하여 정확한 발병률 산출과 결과의 해석이 가능하도록 할 필요가 있다. 또한 유증상자 491명 중 376명(76.6%)이 검사를 받아 (figure 1) 발병률이 낮게 산출되었을 수 있다. 둘째, 식품 섭취력 조사의 응답률이 50.1%로 낮았으며 섭취량이 조사되지 않았다. 식품 섭취량 역시 발병에 주요한 영향을 준다는 점에서 조사가 필요하며 양-반응 관계 분석을 통해 인과성을 평가하는 중요한 부분이 될 수 있다. 향후 조사에서는 응답률 제고를 위해 노력할 필요가 있으며 섭취량도 조사되어야 하겠다. 셋째, 이 조사에서는 카드결제자의 명단만으로 코호트를 구축하였으므로 현금결제자가 누락되었다. 한국은행의 발표자료에 의하면 2019년 식당과 커피숍 이용자의 현금 결제율은 16.4%이고 연령이 증가할수록 현금결제자 비율이 높았다[27]. 현금결제자 누락을 줄이기 위해 현금영수증 발행 명단을 확보할 수 있는 법적인 근거 마련이 필요하다.

우리나라는 20대에서 40대 사이 연령군의 A형간염 IgG 항체양성률이 낮아 감수성 집단의 규모가 크기 때문에 수년 주기로 유행이 반복되고 있다 [10]. 또한 A형간염은 연령이 증가함에 따라 중증도가 증가하므로 [21] 향후 감수성 집단의 연령 증가에 따라 중증도도 증가할 것으로 예상된다. 유행을 조기에 발견할 수 있도록 분자유전학적 감시체계를 확립하고 지역의 역학조사 역량을 강화하여야 한다. 또한 A형간염 바이러스에 오염될 가능성이 있는 식품에 대한 검사도 확대할 필요가 있다.

**Conflict of interest**

The author has no conflicts of interest to declare for this study.

**Acknowledgements**

역학조사를 수행한 부산시 16개 보건소 담당자들에게 깊은 감사를 전합니다.

This work was supported by the Dong-A University research fund.

**References**

1 Martin A, Lemon SM. Hepatitis A virus: From discovery to vaccines. Hepatology 2006;43:S164–72. doi:10.1002/HEP.21052

2 Pinkbook: Hepatitis A | CDC. https://www.cdc.gov/vaccines/pubs/pinkbook/hepa.html (accessed 22 Sep 2021).

3 Chodick G, Ashkenazi S, Lerman Y. The risk of hepatitis A infection among healthcare workers: A review of reported outbreaks and sero-epidemiologic studies. J. Hosp. Infect. 2006;62:414–20. doi:10.1016/j.jhin.2005.07.018

4 Park JY, Lee JB, Jeong SY, et al. Molecular characterization of an acute hepatitis A outbreak among healthcare workers at a Korean hospital. J Hosp Infect 2007;67:175–81. doi:10.1016/J.JHIN.2007.07.020

5 Foster M, Ramachandran S, Myatt K, et al. Hepatitis A Virus Outbreaks Associated with Drug Use and Homelessness — California, Kentucky, Michigan, and Utah, 2017. MMWR Morb Mortal Wkly Rep 2018;67:1208–10. doi:10.15585/mmwr.mm6743a3

6 Halliday ML, Kang LY, Zhou TK, et al. An epidemic of hepatitis A attributable to the ingestion of raw clams in Shanghai, China. J Infect Dis 1991;164:852–9. doi:10.1093/infdis/164.5.852

7 Severi E, Verhoef L, Thornton L, et al. Large and prolonged food-borne multistate hepatitis a outbreak in europe associated with consumption of frozen berries, 2013 to 2014. Eurosurveillance 2015;20. doi:10.2807/1560-7917.ES2015.20.29.21192

8 Donnan EJ, Fielding JE, Gregory JE, et al. A multistate outbreak of hepatitis a associated with semidried tomatoes in Australia, 2009. Clin Infect Dis 2012;54:775–81. doi:10.1093/cid/cir949

9 Wheeler C, Vogt TM, Armstrong GL, et al. An Outbreak of Hepatitis A Associated with Green Onions. N Engl J Med 2005;353:890–7. doi:10.1056/NEJMoa050855

10 Ki M, Son H, Choi BY. Causes and countermeasures for repeated outbreaks of hepatitis A among adults in Korea. Epidemiol Health 2019;41:e2019038. doi:10.4178/epih.e2019038

11 Korea Disease Control and Prevention Agency. Hepatitis A virus detection in salted clams (Press release, 2019JUN25). 2019. https://www.kdca.go.kr/board/board.es?mid=a20501010000&bid=0015&list_no=144301&cg_code=&act=view&nPage=1 (accessed 22 Sep 2021).

12 Korea Disease Control and Prevention Agency. Hepatitis A virus detection in salted clams imported from China (Press release, 2019JUL26). 2019. https://www.kdca.go.kr/board/board.es?mid=a20501010000&bid=0015&list_no=364588&cg_code=&act=view&nPage=1 (accessed 9 Aug 2021).

13 Ministry of Food and Drug Safety. Announcement of hepatitis A virus genetic test results for salted clams (Press release, 2019SEP27). 2019. https://www.kdca.go.kr/board/board.es?mid=a20501010000&bid=0015&list_no=364915&cg_code=&act=view&nPage=1 (accessed 22 Sep 2021).

14 D L. Viruses and bivalve shellfish. Int J Food Microbiol 2000;59:81–116. doi:10.1016/S0168-1605(00)00248-8

15 Polo D, Vilariño ML, Manso CF, et al. Imported mollusks and dissemination of human enteric viruses. Emerg Infect Dis 2010;16:1036–8. doi:10.3201/eid1606.091748

16 Da Silva AK, Le Saux JC, Parnaudeau S, et al. Evaluation of removal of noroviruses during wastewater treatment, using real-time reverse transcription-PCR: Different behaviors of genogroups I and II. Appl Environ Microbiol 2007;73:7891–7. doi:10.1128/AEM.01428-07

17 Lee DY, Chae SJ, Cho SR, et al. Nationwide seroprevalence of hepatitis A in South Korea from 2009 to 2019. PLoS One. 2021;16:e0245162. doi:10.1371/journal.pone.0245162

18 Choe YJ, Son H. The changing gender differences in hepatitis a incidence in South Korea. Vaccine 2020;38:712–4. doi:10.1016/j.vaccine.2019.11.048

19 Hwang T, Shin E, Kim J, et al. Seroprevalence Rate of Hepatitis A in Medical Personnel of the National Medical Center of Korea, 2013-2016. Korean J Fam Pract 2018;8:864–9. doi:10.21215/KJFP.2018.8.6.864

20 Cho K-S, Park S-H. Epidemiologic Study on Hepatitis A Virus Seroprevalence in Busan. Korean J Clin Lab Sci 2014;46:17–21. doi:10.15324/KJCLS.2014.46.1.17

21 Lemon SM. Type A Viral Hepatitis. http://dx.doi.org/101056/NEJM198510243131706 1985;313:1059–67. doi:10.1056/NEJM198510243131706

22 Hu X, Collier MG, Xu F. Hepatitis A Outbreaks in Developed Countries: Detection, Control, and Prevention. Foodborne Pathog Dis 2020;17:166–71. doi:10.1089/fpd.2019.2648

23 Korea Disease Control and Prevention Agency. To prevent hepatitis A, it is recommended to stop consuming salted clams for which safety has not been confirmed (Press release, 2020JUN04). 2020. https://www.kdca.go.kr/board/board.es?mid=a20501010000&bid=0015&list_no=367416&cg_code=&act=view&nPage=1 (accessed 9 Aug 2021).

24 Korea Disease Control and Prevention Agency. Hepatitis A warning due to consumption of imported salted clams (Press release, 2021MAY27). 2021. https://www.kdca.go.kr/board/board.es?mid=a20501010000&bid=0015&list_no=713454&cg_code=&act=view&nPage=1 (accessed 9 Aug 2021).

25 Amon JJ, Devasia R, Xia G, et al. Molecular epidemiology of foodborne hepatitis A outbreaks in the United States, 2003. J Infect Dis 2005;192:1323–30. doi:10.1086/462425

26 Franklin N, Camphor H, Wright R, et al. Outbreak of hepatitis A genotype IB in Australia associated with imported frozen pomegranate arils. Epidemiol Infect 2019;147. doi:10.1017/S0950268818003515

27 Bank of korea. Results of 2019 Payment Methods and Mobile Financial Service Usage Behavior. 2020. http://www.bok.or.kr/portal/bbs/B0000232/view.do?nttId=10056898&menuNo=200725 (accessed 22 Sep 2021).

Figure 1. Study flow of restaurant A visitor cohort study


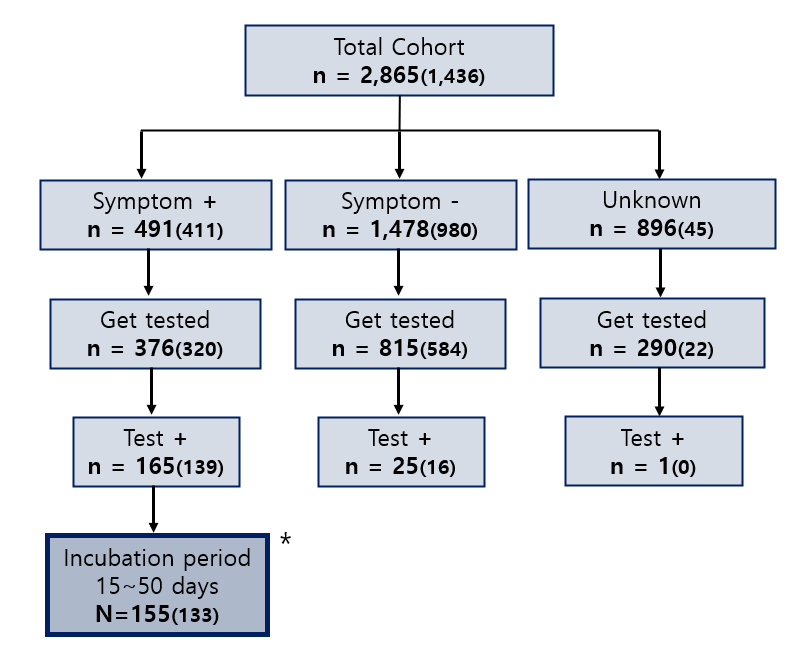


* Eligible for case definition

The numbers in parentheses indicate the number of people who responded to the food intake history

Figure 2. Epidemic curve (B) and distribution of date of visit (A)


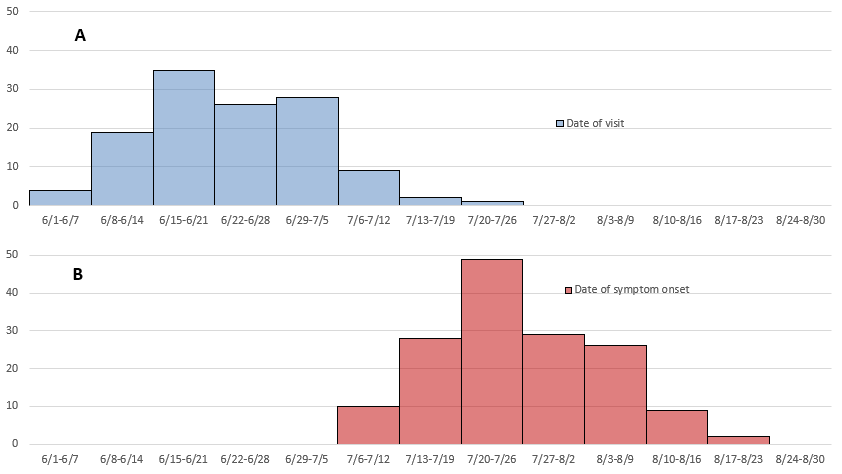


Table 1. Attack rate by age group, gender and visit date

|  |  | Total cohort | Number of cases | Attack rate (%) |
| --- | --- | --- | --- | --- |
| Total |  | 2,865 | 155 | 5.4 |
| Age group | <20 | 68 | 2 | 2.9 |
|  | 20-29 | 555 | 38 | 6.8 |
|  | 30-39 | 886 | 73 | 8.2 |
|  | 40-49 | 495 | 34 | 6.9 |
|  | 50≤ | 283 | 6 | 2.1 |
|  | unknown | 578 | 2 | 0.3 |
| Gender | male | 1,137 | 100 | 8.8 |
|  | female | 924 | 52 | 5.6 |
|  | unknown | 804 | 3 | 0.4 |
| Visit date | 6/1-6/7 | 352 | 6 | 1.7 |
|  | 6/8-6/14 | 326 | 22 | 6.7 |
|  | 6/15-6/21 | 348 | 43 | 12.4 |
|  | 6/22-6/28 | 393 | 31 | 7.9 |
|  | 6/29-7/5 | 380 | 38 | 10.0 |
|  | 7/6-7/12 | 320 | 11 | 3.4 |
|  | 7/13-7/19 | 326 | 3 | 0.9 |
|  | 7/20-7/28 | 379 | 1 | 0.3 |
|  | unknown | 41 | 0 | 0.0 |

Table 2. Relative risk for ingestion of each food item

| Food item | AR of  ingestion group^1^ | | AR of  non-ingestion group^2^ | | RR (95% CI) | |
| --- | --- | --- | --- | --- | --- | --- |
| Pork belly | 7.8 | (98/1,256) | 6.4 | (7/109) | 1.22 | (0.58-2.55) |
| Pork neck | 8.9 | (73/821) | 6.3 | (32/511) | 1.42 | (0.95-2.12) |
| Sliced pork | 9.7 | (62/636) | 6 | (40/669) | 1.63 | (1.11-2.39) |
| Green onion kimchi | 8.3 | (100/1,204) | 3.4 | (5/146) | 2.43 | (1.00-5.86) |
| Leaf Mustard Kimchi | 8.3 | (96/1,153) | 2.7 | (5/187) | 3.11 | (1.28-7.55) |
| Seasoned soybean paste | 8.2 | (99/1,203) | 3.2 | (5/158) | 2.6 | (1.08-6.29) |
| Salted shrimp | 10.9 | (64/587) | 5.3 | (36/677) | 2.05 | (1.38-3.04) |
| Salted clams | 17.3 | (130/752) | 0.2 | (1/556) | 96.12 | (13.48-685.40) |
| Salted guts of hairtail | 11.1 | (68/611) | 5.1 | (33/652) | 2.2 | (1.47-3.28) |
| Sesame oil with salt | 7.6 | (74/978) | 8 | (27/339) | 0.95 | (0.62-1.45) |
| Pickled vegetables | 7.6 | (71/935) | 8.4 | (29/346) | 0.91 | (0.6-1.37) |
| Lettuce | 7.4 | (77/1,044) | 8.8 | (27/306) | 0.84 | (0.55-1.27) |
| Sesame leaf | 7.7 | (80/1,038) | 7.8 | (24/308) | 0.99 | (0.64-1.53) |
| Pepper | 7.7 | (74/955) | 7.1 | (28/397) | 1.1 | (0.72-1.67) |
| Garlic | 7.2 | (76/1,057) | 8.9 | (26/292) | 0.81 | (0.53-1.24) |
| Boiled rice | 6.7 | (61/913) | 9.9 | (42/425) | 0.68 | (0.46-0.98) |
| Kimchi stew | 8.7 | (72/824) | 5.5 | (27/489) | 1.58 | (1.03-2.43) |
| Soybean paste stew | 5.9 | (21/357) | 8.3 | (79/948) | 0.71 | (0.44-1.12) |
| Spicy noodles | 6.7 | (6/90) | 7.9 | (98/1,247) | 0.85 | (0.38-1.88) |
| Water | 7.5 | (90/1,205) | 9.6 | (14/146) | 0.78 | (0.46-1.33) |

^1^ % (No. of cases/No. of people who ingested)

^2^ % (No. of cases/No. of people who did not ingest)

AR, attack rate; RR, relative risk; CI, confidence interval

Table 3. The association between food consumed and Hepatitis A cases

|  |  | aOR | 95% CI |
| --- | --- | --- | --- |
| Age group | 0~29 | ref. |  |
|  | 30~39 | 1.53 | (0.86-2.70) |
|  | 40~49 | 1.05 | (0.54-2.04) |
|  | 50 and over | 0.37 | (0.11-1.18) |
| Gender | Male | ref. |  |
|  | Female | 0.49*** | (0.30-0.80) |
| Visit date | 6/1~6/7 | ref. |  |
|  | 6/8~6/14 | 2.77 | (0.94-8.18) |
|  | 6/15~6/21 | 9.24*** | (3.34-25.55) |
|  | 6/22~6/28 | 3.40** | (1.20-9.63) |
|  | 6/29~7/5 | 4.74*** | (1.71-13.09) |
|  | 7/6~7/12 | 1.29 | (0.32-5.13) |
|  | 7/13~7/19 | 0.38 | (0.04-3.42) |
|  | 7/20~7/28 | 0.45 | (0.05-4.12) |
| Seasoned soybean paste | No | ref. |  |
|  | Unkonwn | 7.92** | (1.31-47.89) |
|  | Yes | 2.55 | (0.93-7.02) |
| Salted shrimp | No | ref. |  |
|  | Unkonwn | 0.70 | (0.22-2.21) |
|  | Yes | 0.70 | (0.43-1.14) |
| Salted clams | No | ref. |  |
|  | Unkonwn | 5.10 | (0.27-96.43) |
|  | Yes | 68.62*** | (9.22-510.87) |
| Sliced pork | No | ref. |  |
|  | Unkonwn | 1.43 | (0.48-4.31) |
|  | Yes | 1.62** | (1.01-2.60) |
| Boiled rice | No | ref. |  |
|  | Unkonwn | 0.41 | (0.12-1.42) |
|  | Yes | 0.52*** | (0.32-0.84) |

*p<.05, ** for p<.01, and *** for p<.001

aOR, adjusted Odds Ratio; CI, Confidence Interval
